# Prevalence and patterns of substance use in conflict-affected settings: findings of a cross-sectional study from south-central Somalia

**DOI:** 10.3389/fpsyt.2025.1729539

**Published:** 2026-01-22

**Authors:** Mohamed Ibrahim, Abdulwahab M. Salad, James Mwangi Ndithia, Kato Francis, Zeynab Noor, Mohamed Aden Hillow, SK Md Mamunur Rahman Malik

**Affiliations:** 1 School of Social Work, University of British Columbia, Vancouver, British Columbia, Canada; 2 World Health Organisation, Somalia Country Office, Mogadishu, Somalia; 3 School of Public Health and Tropical Medicine, Somali National University, Mogadishu, Somalia; 4 Department of Mental Health and Substance Use, Federal Ministry of Health, Mogadishu, Somalia; 5 Department of Psychiatry, Wajir County Referral Hospital, Wajir, Kenya; 6 World Health Organization, Eastern Mediterranean Regional Office, Cairo, Egypt

**Keywords:** conflict, conflict-affected population, internally displaced persons, prevalence, Somalia, sub-Saharan Africa, substance use, youth

## Abstract

**Introduction:**

The south-central region of Somalia has faced recurrent armed conflicts, unrest and climatic shocks resulting in forced displacement, marginalization and social exclusion of the people affected by these enduring humanitarian crises. While psychological trauma and economic hardships are recognized contributors to mental health conditions and substance use, evidence on the prevalence and patterns of substance use in Somalia remains scarce. This study investigates the prevalence and patterns of substance use among populations living in conflict-affected districts of South-Central Somalia.

**Methods:**

A community-based cross-sectional study was conducted among 694 participants who were selected from three purposively selected conflict-affected districts of south-central Somalia using a multi-stage systematic random sampling procedure with probability proportion to size (PPS) technique. The study participants belonged to both host communities and living in the camps of internally displaced people (IDP). All people, both male and female aged ≥18 years living in these districts for at-least 6 months preceding the study were eligible for inclusion in the study. The study was conducted from October to December 2021. A specially designed questionnaire was used for collecting the data on socio-demographic characteristics, while the WHO Alcohol, Smoking, and Substance Involvement Screening Test (ASSIST) tool was used for collection of data on the use of psychoactive substances. Descriptive and inferential analyses were performed, and logistic regression was applied to identify socio-demographic determinants of substance use.

**Results:**

The overall lifetime prevalence of any substance use among the study participants was 53.60% (95% CI: 49.89%-57.31%) and the prevalence of current substance use was 51.30% (95% CI: 47.58%-55.02%). Among lifetime users, tobacco was the most commonly used substance (39.19%), followed by sedatives (37.46%), while cocaine was the least used (0.72%) substance. Males had higher odds of lifetime substance use compared to females (aOR = 7.31; 95% CI: 5.09–10.50). Participants who studied in Madrasah/Quranic school were less likely to have lifetime substance use compared to those with higher education (aOR = 0.29; 95% CI: 0.14–0.57). Those who were single showed significant association with lifetime substance use compared to married individuals (aOR=1.94; 95% CI: 1.34–2.79).

**Conclusion:**

High prevalence of both lifetime and current illicit substance use was identified in young male people living in the conflict-affected areas of Somalia. These findings highlight the need for integrated public health and regulatory responses to address substance use conflict-affected Somali populations, alongside efforts to strengthen treatment capacity.

## Introduction

Mental health and substance use disorders are major public health consequences of protracted conflicts and humanitarian crises across the world (1) and have been highlighted as one of the 10 key public health challenges for 2024 (2). Recent research has shown that one in every five people (22%) in post-conflict settings experience common mental health conditions such as depression, anxiety, post-traumatic stress disorder, bipolar disorder, or schizophrenia driven by factors including loss of livelihood, forced displacement, hunger, poverty and social exclusion (3). Substance use and mental health disorders are often interconnected, and substance use disorders often co-occur with mental health conditions exacerbating poor health outcomes, including all-cause premature mortality and suicides (1, 4, 5).

In conflict and humanitarian crisis settings, affected individuals are at greater risk of developing common mental health conditions such as post-traumatic stress disorder (PTSD), depression, and anxiety, which in turn increases the likelihood of substance use (4, 6, 7). In such conflict-affected settings, especially during large-scale civil wars, the collapse of government institutions, disruption of public services, and weakening of regulatory systems can further facilitate the proliferation of illegal and counterfeit drug trade and contribute to substance use among individuals facing economic hardships, unrest, war and psychological trauma. Often people resort to harmful practices such as substance use in such environments as a coping mechanism against economic hardships, uncertain future, stressful environment, and loss of livelihoods in conflict settings (8, 9). The prolonged duration of the conflicts is also known to increase the vulnerability of the affected people to substance use. A systematic review has shown that substance use, especially use of alcohol, have been rampant in such situations where people are exposed to multiple stressors such as trauma, forced displacement, physical and sexual violence, loss of loved ones and livelihoods (10).

The World Health Organization (WHO) defines psychoactive substances as substances which, when consumed or administered, have the ability to change consciousness, mood or thinking processes (1). Though, what is considered as “psychoactive” are sometimes difficult to delineate clearly, there is a common recognition that alcohol, nicotine, opioids, cannabis, cocaine, amphetamines and other stimulants, hallucinogens, hypnotics and sedatives, are recognized as psychoactive substances. The analysis of data on the use of psychoactive substances shows that an estimated 5.5% of the adult populations (i.e., those aged 15–64 years) used illicit drugs in 2022 (11). Whereas, in the Eastern Mediterranean Region of WHO, an estimated 6·7% of the adult population (i.e., those aged 15–64 years) used any illicit drug in 2022, corresponding to approximately 30 million people with males having higher prevalence (10.9%) compared to females (2.5%) (12). Data on mental and substance use disorders published by the Institute of Health Metrics and Evaluation in 2019 showed that the worldwide prevalence of substance-use disorders was 2.2%, with higher prevalence of alcohol-use disorders (1.5%) than other drug-use disorders (0.8% total including: cannabis 0.32%; opioid 0.29%, amphetamine 0.10%; cocaine 0.06%) (13).

Somalia has experienced more than three decades of civil war, political instability and climate-related emergencies leading to widespread poverty, mass and forcible internal displacement and economic hardships for its people living in conflict-affected settings. Its humanitarian crisis is one of the most enduring crises in Africa. The conflict and violence, exacerbated by recurrent climactic shocks, have fragmented the social fabrics of the country, eroding community resilience and coping mechanisms especially of its youth. In 2022, during the peak of severe drought and food insecurity situation, Somalia had over 2.6 million internally displaced people (IDP) living in extreme hardship or catastrophic conditions, dependent on humanitarian assistance for survival (14). The ongoing conflict, insecurity and prolonged humanitarian crisis, for decades, have resulted in a large number of people, especially the youth, suffering from multilayered mental health conditions. A study conducted jointly by the Federal Government of Somalia and WHO in three conflict-affected zones of Somalia showed high prevalence of common mental health conditions (78.1%), with anxiety disorders being the commonest (15).

Overall, data on substance use among conflict-affected populations in Somalia remain limited. Historically, khat has been widely used in many parts of the country and is viewed as a traditional recreational stimulant commonly consumed by elders during special occasions such as weddings and religious ceremonies (16–18). More recently, however, media reports and stakeholder accounts from the health and humanitarian sectors have raised concerns about increasing use of prescription drugs—including benzodiazepines and tramadol—particularly among youth in Mogadishu (19). This emerging pattern of psychoactive substance use has been linked to the collapse of state institutions and large-scale displacement, which have contributed to the wider availability and accessibility of alcohol, cannabis, and illicit street drugs, especially among young people across Somalia (19, 20).

In the absence of population-based epidemiological data on the prevalence and patterns of substance use in Somalia, and recognizing this key research gap, the current study was undertaken in three conflict-affected districts of south-central part of Somalia, which hosts large numbers of people who are internally displaced by conflict. The purpose of the study was to determine the prevalence and epidemiological patterns of substance use and study its socio-demographic determinants among the populations living in these conflict-affected districts of Somalia. As substance use and mental health disorders are often interconnected, and we have found high prevalence of common mental health conditions (78.1%), with anxiety disorders being the commonest in one of our previous studies (15), we presumed that prevalence of substance use among the conflict-affected youth would also be high.

## Methodology

### Study design, setting and period

This was a community-based cross-sectional study conducted among participants residing in three districts—Baidoa, Dolow, and Kismayo—located in the south-central region of Somalia, within the Southwest and Jubaland States. These districts, situated approximately 240 to 500 kilometers from Mogadishu, the capital city, were purposively selected due to their continued exposure to active conflict during the study period and their protracted experiences with drought, famine, and large-scale internal displacement resulting from war, inter-clan violence, and climate-related emergencies. The selected areas also host significant populations of returnees and former combatants. Data collection was carried out between October and December 2021.

### Study population, inclusion and exclusion criteria

All people, both male and female aged ≥18 years living in the study areas at least six months preceding the study and belonging to either the host communities or Internally Displaced Persons (IDPs) were eligible for being selected for the study. Participants who had serious health conditions or disabilities that impeded their ability to competently consent and or complete the questionnaire were excluded from the study.

### Sample size determination

The sample size was estimated using multistage sampling procedure. We used the estimated prevalence of substance use not exceeding 33% based on a literature search on prevalence of substance abuse in conflict-affected settings (21). Using single population proportion formula, the sample size was calculated with the confidence level of 95% and margin of error of 5%. We have added a contingency of 10% for the non-response rate and estimated the sample size to be 340. Since the sampling was multistage, a design effect of 2 was added and as such the final total sample size was estimated to be 680 from three study sites.

### Sampling procedure

We used multistage systematic sampling procedure with probability proportion to size (PPS) technique to select the study participants. After purposely selecting the three study sites, we calculated the proportion of minimum sample size that should be selected from each of three study sites. We have considered the estimated population of each of these study sites which included both host communities and internally displaced persons. We have used the population estimates and age structures of these sites from the Somali Health and Demographic Survey 2020 (22) which was the most recent nationwide population estimation undertaken in Somalia at the time of study.

After determining the minimum sample size from each of three study sites, we counted the total number of households (for host communities) and camps (for IDPs) in each of these sites and using the population size and age structure, we determined the number of households that would be required to be covered for the minimum sample size. We, then calculated the interval (K) by dividing the number of households by the sample size allocated to each study site. After the first household was selected randomly, every K value (10) was used to select the next household by adding 10 to the first household. In the final stage, all household members meeting our inclusion criteria were invited to participate in the study. In case of refusal, next household was visited. This process continued until the minimum sample size for each study site was reached. While the minimum sample size required for the study was 680, we recruited 694 participants from three districts to increase the power of the study and also taking into consideration non-response rate.

### Study instruments

We used the WHO Alcohol, Smoking, and Substance Involvement Screening Test (ASSIST) tool which is a brief screening questionnaire developed and validated by WHO for collecting data on substance use in low and middle-income countries (23). Through its eight items, we collected information on and determine levels of risk from the use of tobacco, alcohol, cannabis, amphetamine-type stimulants, cocaine, sedatives, hallucinogens, opioids, and other drugs. We have also used a researcher-designed questionnaire to collect sociodemographic information such as: IDPs or living in the community; sex; age group; marital status; dwelling place; level of education; employment status and household size. The Somali National University was involved in developing this tool to ensure sociocultural relevance. We collected information on substances identified as “psychoactive substances” by WHO, which, when consumed or administered, have the ability to change consciousness, mood or thinking processes.

### Data collection procedure and quality control

Data were collected between 25 October and 15 November 2021 through a face-to-face interview using a pre-tested, structured, and standardized questionnaire and administered via the Kobo Toolbox mobile application. This questionnaire, applied in Somali language, was used to collect socio-demographic characteristics data of the study participants. The WHO ASSIST tool was used to collect data on substance use which was adapted to the Somali context by three authors of this paper (a mental health specialist, a psychologist, and a public health specialist) all of Somali origin. The adaptation ensured that the instrument was culturally and linguistically appropriate for Somali context. The questionnaire was piloted prior to full implementation to test its clarity and feasibility. A total of 60 enumerators (20 per site), all holding at least a bachelor’s degree, were involved in data collection. They were also trained on the collection of data using the questionnaire and WHO ASSIST tool. Data on socio-demographic characteristics were collected electronically using KoBoCollect with Global Positioning System (GPS) integration to record the physical location of interviews and ensure data integrity. All data were anonymized and securely stored. All data were checked for completeness, accuracy and consistency before submission almost on every day after the data collection.

### Study variables and data analysis

Lifetime substance use was considered the dependent variable while independent variables included socio-demographic factors such as age, sex, marital status, education level, family size, occupation and area of residence. The collected data were checked for completeness and consistency, then edited and coded using the Kobo toolbox application and exported to STATA (Version 18) statistical software for analysis. Descriptive statistics, including frequency and percentage, were calculated to explain the socio-demographic characteristics and substance use related factors. Crude unweighted prevalence rates with 95% confidence intervals (CI) were calculated for both lifetime and current (3 months) use of substance, while crude prevalence rates for each category of substance were calculated for both lifetime and current use to determine factors associated with lifetime substance use, bi-variate analysis was performed to ascertain the association between sociodemographic characteristics and ASSIST diagnoses. An association was considered statistically significant if the p-value level was less than or equal to 0.05 (p ≤ 0.05). Subsequently, significant variables in the bi-variable analysis (value of p <0.05) were further included in the multi-variable logistic regression model, and independent associations were examined by calculating both crude and adjusted odds ratio, considering the following variables: age, gender, educational level, household size, area of residence, occupation status, marital status, and study site. Statistical significance was declared at p < 0.05 and with corresponding 95% confidence intervals. Chi-square tests were used to analyze categorical variables and assess differences in substance use proportions across socio-demographic characteristics.

### Ethical considerations

The Somali National University Institutional Review Board has approved the study. Written informed consent was obtained from all participants, who were informed of their right to withdraw from the study at any time without loss of benefits such as access to research report the individual interviews took place in a private environment to protect privacy. All questionnaires were encrypted for confidentiality, and the codes were kept by a designated research team member. No personal identifiers were entered into the data platform, nor was the information accessible to unauthorized personnel.

## Results

### Socio-demographic characteristics

The sociodemographic characteristics of the participants are shown in **Table 1**. We recruited a total of 694 participants for this study (Baidoa-208, Dollow-230 and Kismayo-256). More than half of the participants were male (58.50%). The participants’ mean age was 32.60 (± 10.70) years. The majority of participants were aged 20–33 years (61.24%), corresponding to the youth in Somalia. The largest proportion of participants were married (51.15%), illiterate (41.21%), unemployed (86.74%), and lived in households with more than 5 members (63.83%) at the time of study. Only 19.45% of study participants were living in the IDP camps while the majority were from host communities (80.55%).

**Table 1:** Sociodemographic characteristics of the respondents, Somalia, 2021 (n=694).

Variable (n=694)	Frequency (%)
Age group	
20-33	425 (61.24)
34-46	186 (26.80)
47-60	83 (11.96)
Sex	
Male	406 (58.50)
Female	288 (41.50)
Educational status	
Illiterate	286 (41.21)
Madrasah/Quran	267 (38.47)
Secondary or less	92 (13.26)
University	49 (7.06)
Marital status	
Single	205 (29.54)
Divorced/widowed	134 (19.31)
Married	355 (51.15)
Occupation	
Unemployed	602 (86.74)
Employed	92 (13.26)
Family size	
1–3 Members	75 (10.81)
4–5 Members	176 (25.36)
>5 Members	443 (63.83)
Area of residence	
Community	559 (80.55)
IDP camp	135 (19.45)
Area of the study	
Kismayo	256 (36.89)
Baidoa	208 (29.97)
Dolow	230 (33.14)

### Prevalence of substance use over time

**Figure 1:**
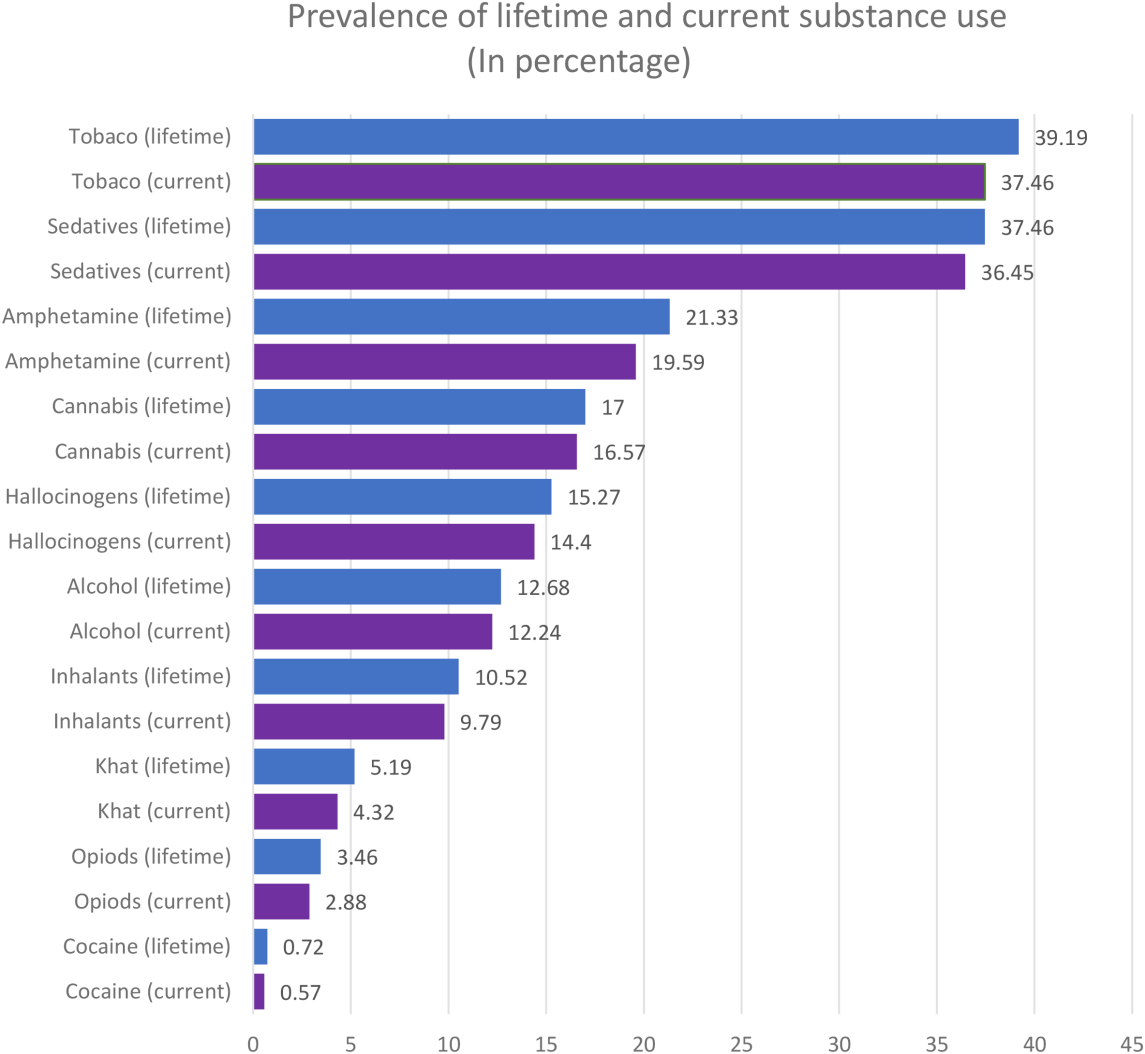
Prevalence of lifetime and current substance use (In percentage).

**Figure 1** summarizes the lifetime and current (past three months) use, as measured by the WHO ASSIST tool. Substances are presented in descending order of frequency. The overall prevalence of lifetime substance use was 53.60% (95% CI: 49.89%-57.31%), and the prevalence of current substance use was 51.30% (95% CI: 47.58%-55.02%).

Tobacco was the most commonly used substance, with a lifetime use of 39.19% followed closely by sedatives with a lifetime use of 37.46%, while cocaine was the least used (0.72%).

### Socio-demographic characteristics associated with substance use

A wide variation was observed between different sociodemographic groups and various substances used. **Table 2** summarizes the bivariate analyses showing crude associations between socio-demographic factors and lifetime substance use (p<0.05). Significant variations were observed in substance use disorders by gender, community type (IDP or host), and study site, with all differences statistically significant (p<0.05).

**Table 2:** Prevalence of substance use (lifetime) by socio-demographic characteristics (n=694).

Independent variables	Substance use (lifetime)		P-value #
Variable	Yes, n (%)	No, n (%)	
Age			
20-33	249 (66.94)	176 (54.66)	0.000*
34-46	97 (26.08)	89 (27.64)	
47-60	26 (6.99)	57 (17.70)	
Sex			
Male	297 (79.84)	109 (33.85)	0.000*
Female	75 (20.16)	213 (66.15)	
Education			
Illiterate	177 (47.58)	109 (33.85)	0.000*
Madrasah/Quran	108 (29.03)	159 (49.38)	
Secondary or less	52 (13.98)	40 (12.42)	
University	35 (9.41)	14 (4.35)	
Marital Status			
Single	131 (35.22)	74 (22.98)	0.002*
Divorced/widowed	66 (17.74)	68 (21.12)	
Married	175 (47.04)	180 (55.90)	
Occupation			
Unemployed	303 (81.45)	299 (92.86)	0.000*
Employed	69 (18.55)	23 (7.14)	
Family size			
1–3 members	33 (8.87)	42 (13.04)	0.189
4–5 members	99 (26.61)	77 (23.91)	
	240 (64.52)	203 (63.04)	
Area of residence			
Community	335 (90.05)	98 (30.43)	0.000*
IDP camp	37 (9.95)	224 (69.57)	

**#**Result of χ2 test.

IDP, Internally Displaced People.

*statistically significant at P < 0.05.

In the bivariate analysis, males had a significantly higher lifetime substance use than females (79.84% vs 20.16%; p<0.05). Participants aged 20-33 (66.94%), who were illiterate (47.58%), married (47.04%), unemployed (81.45%), and living in the host communities (90.05%) had significantly (p<0.05) higher substance use proportions compared to other corresponding groups in the covariates.

There was no statistically significant difference in lifetime substance use with family size of the participants (p=0.189).

### Determinants of lifetime substance use

In the unadjusted logistic regression analysis (**Table 3**), males had increased likelihood of using substance (lifetime) than females (OR = 7.73; 95% CI:5.49-10.90). In addition, participants aged 20–33 years (OR = 3.10; 95% CI:1.87-5.12), those who were single (OR = 1.82; 95% CI: 1.27-2.59), and those living in the host communities (OR = 3.96; 95% CI: 2.61-5.99) had higher odds and increased likelihood of using substance (lifetime). When the effects of covariates were controlled for in the multivariate logistic regression analysis, only males, and those who were single and living in the community remained significantly associated with lifetime substance use. Males had increased odds of substance use than females (aOR=7.31; 95% CI: 5.09-10.50). Those who were single also had increased odds compared to participants who were married or divorced (aOR=1.94; 95% CI: 1.34-2.79), while individuals living in the community had increased odds relative to those who lived in the IDP camps (aOR=3.40; 95% CI: 2.23-5.20). We have also seen in our multivariate logistic regression analysis that those who studied in Quranic school or Madrasah (aOR=0.29; 95% CI: 0.14-0.57), were unemployed (aOR= 0.38; 95% CI: 0.23-0.64), or had studies at a university level at the time of conducting the study were less likely to be associated with lifetime substance use compared to their counterparts.

**Table 3:** Sociodemographic factors associated with substance use (lifetime), Somalia, 2021.

Independent variables	Crude OR (95% CI)	Adjusted OR (95% CI) ^a^
Age		
20-33	3.10 (1.87, 5.12)	1.07(0.55, 1.59)
34-46	2.38 (1.38, 4.12)	0.89 (0.32, 1.46)
47-60	1.00	1.00
Sex		
Male	7.73 (5.49,10.90)	7.31 (5.09, 10.50) *
Female	1.00	1.00
Education		
Illiterate	0.64 (0.33, 1.26)	065 (0.33,1.27)
Madrasah/Quran	0.27 (0.13,0.52)	0.29(0.14, 0.57) *
Secondary or less	0.52 (0.24,1.09)	0.50(0.23,1.07)
University	1.00	1.00
Occupation		
Unemployed	0.33 (0.20, 0.55)	0.38 (0.23,0.64) *
Employed	1.00	1.00
Marital Status		
Single	1.82 (1.27,2.59)	1.94 (1.34,2.79) *
Divorced/widowed	0.99 (0.67,1.48)	1.10 (0.73,1.65)
Married	1.00	1.00
Family size		
1–3 Members	0.66 (0.40, 1.08)	0.66 (0.40,1.10)
4–5 Members	1.08 (0.76, 1.54)	1.05 (0.73, 1.50)
>5 Members	1.00	1.00
Area of residence		
Community	3.96 (2.61,5.99)	3.40 (2.23,5.20) *
IDP camp	1.00	1.00

*Statistically significant in the multivariate analysis (P<0.05).

a Adjusted for covariates.

## Discussion

This study represents the largest community-based investigation conducted in conflict-affected populations of Somalia to examine the prevalence and patterns of substance use, encompassing all psychoactive substances listed by the WHO. To our knowledge, this provides the most recent evidence on substance use within these populations. Overall, 53.60% of study participants reported lifetime substance use, and 51.30% of study participants reported current use. Substance use was widespread among conflict-affected groups, with tobacco, sedatives and amphetamines being the three most commonly used substances for both lifetime and current use. Male participants, those aged 20–30 years, individuals with single marital status, and those living in host communities were more likely to report lifetime substance us. Conversely, participants who studied in Quranic school or Madrasah, and those who were unemployed had lower association with lifetime substance use.

In the absence of previous studies on substance use in Somalia, direct comparisons or trend analysis were not possible. However, our findings on substance use patterns align with those reported in neighboring countries in the recent past. For example, in Ethiopia (9) a study conducted in 2023 found an overall lifetime prevalence of substance use as 48.10% and that of current substance use as 72.50%. Among lifetime users, highest prevalence was noted for khat (76.5%), alcohol (49.00%), various forms of tobacco (33.30%) and cannabis (23.00%). Similar to our findings, being male and single, having a family history of substance use, low perceived social support, and the presence of mental health conditions were associated with an increased likelihood of substance use in Ethiopia. In Kenya, on the other hand, the prevalence of substance use was found to be 86.00%, exceeding both Ethiopia and Somalia. In line with our findings, prevalence rates in Kenya were higher in males and those who were single, with alcohol, tobacco and khat reported as the most frequently used substance (24).

The findings of a systematic review and meta-analysis done on observational studies and conducted among the youth in Sub-Sharan Africa (SSA) shows the pooled lifetime and current prevalence of any substance use as 21.00% and 15% respectively (25). Though this overall lifetime prevalence, drawn from a pooled analysis of 53 studies, is lower than what our study has found out, this meta-analysis also showed that there was a wide variation in both the lifetime and the current prevalence of substance use. For example, the prevalence of lifetime substance use varied from 2% in a study from Ethiopia to as high as 56.0% in Nigeria. Similarly, the current prevalence of any substance use varied from 2.5% in one study to as high as 75.0% in another study. Though not similar to our findings, alcohol use problem was the most prevalent (40%), followed by khat use (25%), stimulant use (20%), and cigarette smoking (16%) among the youth in the SSA. Similar to our study findings, the prevalence of substance use problems was found higher among male youth with negative family dynamics. The findings of this meta-analysis are also aligned with our study findings indicating that those attending religious school (regardless of the type of religion) was associated with a lower risk of substance use among youth while being single was associated with higher odds of substance use.

The findings from our study show similar types of substance use observed in other conflict-affected countries, though most of these study findings are not from population-based studies or have not been conducted in recent time. In Afghanistan, a 2015 study found that over 11% of the population were using psychoactive drugs, and the country had the highest prevalence rate of opioid use in the world at that time (26). In Yemen, with an ongoing war and conflicts, no nationally representative data on substance use are available. However, several studies have reported increased consumption of Khat as well as other illicit drugs such as methamphetamine and cocaine (27). A study from the West Bank done in 2016 showed that substance use, such as energy drinks, tobacco, alcohol and other illicit drugs, are high among the Palestinian teenagers living in the city, villages or refugee camps with significantly higher prevalence of each substance among those living in the refugee camps in the West Bank (28). In Sri Lanka, a study conducted in a post-conflict setting found that about 7% of participants aged 15–19 years reported using substances, with the majority being male users who reported tobacco, alcohol, and other illicit substances (29), similar to our findings. In South Sudan, a country with a prolonged history of civil war, over 14% of participants in a study were identified as harmful or hazardous drinkers of alcohol (30). Being male, experiencing financial instability, and psychological distress were the main risk factors for alcohol misuse among these study participants. In Ukraine, another war-affected country, alcohol was the most prevalent substance used as a coping mechanism with overall prevalence of 3.20% -8.40% for men and 0.70% for women (31). When restricted to current drinkers the prevalence was present in 14.30% of men and 1.70% of women studied. Another study conducted in Ukraine among the university students who have experienced trauma, loss of family members and friends after the war in 2022, showed a high prevalence of substance use (66% alcohol use; 11.50% other psychoactive substances) in 2023 (32).

Although our study findings were similar to what other researchers have found out in conflict-affected populations and also in countries of SSA, the patterns of substance use, particularly the widespread use of tobacco, alcohol, and sedatives among the younger population in South-Central Somalia, suggest a potential cultural shift in substance use behavior. This shift appears most pronounced among the younger generation, who have grown up during a prolonged period of conflict. Notably, our study found a lower prevalence of khat among the conflict-affected youth compared to other substances, marking a departure from the findings of earlier studies (2005–2009) that identified khat as the most commonly used substance in Somalia (16–18, 33). The low prevalence of alcohol among the substance users (both lifetime and current users) compared with other substance and when compared with the findings of other countries in SSA is not surprising given the restriction by the Muslim religion that is predominant in Somalia. Cocaine and opioids were the least used, as has been found in studies in the countries surrounding Somalia (9, 24). We have found tobacco as the most used substance in our study populations since its use is not controlled or regulated in the country, and we also understand that sedatives are available widely and anyone can buy sedatives without any prescription in Somalia. It is plausible that the use of sedatives among youth represents a form of self-medication to cope with the cumulative stress arising from ongoing conflict, climate-related disruptions, and economic instability. Moreover, given the increasing prevalence of amphetamine use, sedatives may also be employed as depressant agents (“downers”) to mitigate or counterbalance the heightened effects associated with stimulant consumption (“uppers”).

The growing trend of substance uses among Somali youth, who constitute the majority of the Somali population, is a major public health concern. In our previous study conducted at the same three districts of South-Central Somalia, we observed a high prevalence of anxiety and post-traumatic disorders (15). Many youths have directly experienced or witnessed violence, displacement, and loss, contributing to substance use as a coping mechanism against psychological trauma and daily stressors.

Overall, our findings reveal a widespread use of psychoactive substances such as the tobacco, sedatives, amphetamines, cannabis, and alcohol among the younger generation which indicate emerging trends and can signify a fundamental shift in drug use patterns in Somalia which was historically known for khat use in the past. This highlights the urgent need to develop a more robust framework for the prevention, promotion, and treatment of substance-related conditions in the country.

### Strength and limitation

This is the first community-based study conducted in Somalia among conflict-affected populations. A major strength of this study lies in its ability to capture the prevalence and patterns of substance use (both lifetime and current use), which are included in the WHO’s list of psychoactive substance. This makes our findings comparable with other studies that used the same assessment tool (WHO ASSIST). We also investigated the socio-demographic determinants of lifetime substance use for the study populations which can be helpful, in future, for shaping appropriate policies and for targeting appropriate interventions for appropriate substance user group. The methodology of our study was rigorous. We employed face-to-face interviews of our study participants; our data collectors were adequately trained in study protocol and interview techniques; we used a standard questionnaire and piloted the data collection procedure before fieldwork to ensure validity. Using an online data collection tool with GPS improved data accuracy and reduced inconsistencies by allowing for cross-checking and validation. The diversity of study participants (by gender and other sociodemographic characteristics) and the high response rate (97.80%) further enhanced the reliability and representativeness of our findings. As data were collected from three conflict-affected districts, this approach helped to minimize selection bias and strengthen the generalizability of our study findings. A major limitation of this study is that, due to its cross-sectional design, causal relationships between variables could not be established. Additionally, we cannot fully eliminate the potential for self-reporting bias, which may have been influenced by social desirability or other factors. Finally, as the study was conducted specifically in South-Central Somalia, the findings provide a context-specific snapshot of substance use patterns among conflict-affected populations, which may not entirely reflect substance use patterns in rest of the country.

## Conclusion and recommendations

Our study highlights critical insights into the use of psychoactive substance among conflict-affected populations in South-central Somalia. The findings indicate a concerning trend of substance use among youth living in the conflict-affected areas. Given that youth constitute the majority of Somalia’s population, they have the potential to play a crucial role in the nation’s recovery from decades of conflicts, war, and instability, to serve as agents of change contributing to economic growth and community rebuilding. At the same time, when deprived of education and economic opportunities and while being exposed to continuous violence, youth can be less able to contribute to resilience and reconciliation in their communities. In addition, they are at risk of being exploited in partisan dynamics that can fuel tensions and lead to conflicts. As a result of conflicts, intra-clan violence and civil disruptions experienced in Somalia over the past three decades, much of its population especially the youth are dealing with substance use and other multi-layered psychosocial problems that have never been addressed.

To address these challenges, targeted community-based interventions are urgently needed. A comprehensive policy response, coupled with strong social support systems, will be vital in mitigating the growing threat of substance use in Somalia. The study reaffirms the need to integrate substance use and addiction services into healthcare and social service systems, particularly in conflict and post-conflict contexts. We call for a comprehensive and integrated approach to mental health in Somalia that includes substance use and addiction services across care continuums. These initiatives will represent a crucial move toward addressing the complex interplay of mental health conditions and substance use in challenging environments like that of Somalia. It is also critical to frame substance use as a public health and social issue, rather than one of moral failure or criminality. Compassionate, trauma-informed care should be prioritized over punitive measures, fostering rehabilitation rather than stigma. Ultimately, the findings of this study underscore the urgency of establishing sustainable prevention, treatment, and recovery frameworks to respond to the country’s evolving public health needs.

## Data Availability

The raw data supporting the conclusions of this article will be made available by the authors, without undue reservation.
